# Emerging theragnostic radionuclide applications for hepatocellular carcinoma

**DOI:** 10.3389/fnume.2023.1210982

**Published:** 2023-10-25

**Authors:** N. E. Nyakale, C. Aldous, A. A. Gutta, X. Khuzwayo, L. Harry, M. M. Sathekge

**Affiliations:** ^1^ Department of Nuclear Medicine, Sefako Makgatho Health Sciences University, Dr George Mukhari Academic Hospital, Pretoria, South Africa; ^2^ Department of Nuclear Medicine, University of Kwa-Zulu Natal, Durban, South Africa; ^3^ School of Clinical Medicine, College of Health Sciences, University of KwaZulu-Natal, Durban, South Africa; ^4^ Department of Nuclear Medicine, University of Pretoria, Steve Biko Academic Hospital, Pretoria, South Africa; ^5^ Nuclear Medicine Research Infrastructure (NuMeRI), Steve Biko Academic Hospital, Pretoria, South Africa

**Keywords:** transarterial radionuclide therapy, radioembolization, hepatocellular carcinoma, prostate specific membrane antigen, fibroblast activation protein inhibitors, lipiodol, microspheres, theragnostics

## Abstract

Hepatocellular carcinoma (HCC) is a major global health problem. Theragnostic is a term that refers to the integration of diagnostic and therapeutic modalities into a single system for personalized medicine. Theragnostic care in HCC involves the use of imaging techniques to diagnose the cancer and assess its characteristics, such as size, location, and extent of spread. Theragnostics involves the use of molecular and genetic tests to identify specific biomarkers that can help guide treatment decisions and, post-treatment, assess the dosimetry and localization of the treatment, thus guiding future treatment. This can be done through either positron emission tomography (PET) scanning or single photon emission tomography (SPECT) using radiolabeled tracers that target specific molecules expressed by HCC cells or radioembolization. This technique can help identify the location and extent of the cancer, as well as provide information on the tumor's metabolic activity and blood supply. In summary, theragnostics is an emerging field that holds promise for improving the diagnosis and treatment of HCC. By combining diagnostic and therapeutic modalities into a single system, theragnostics can help guide personalized treatment decisions and improve patient outcomes.

## 1. Introduction

**Figure 1:**
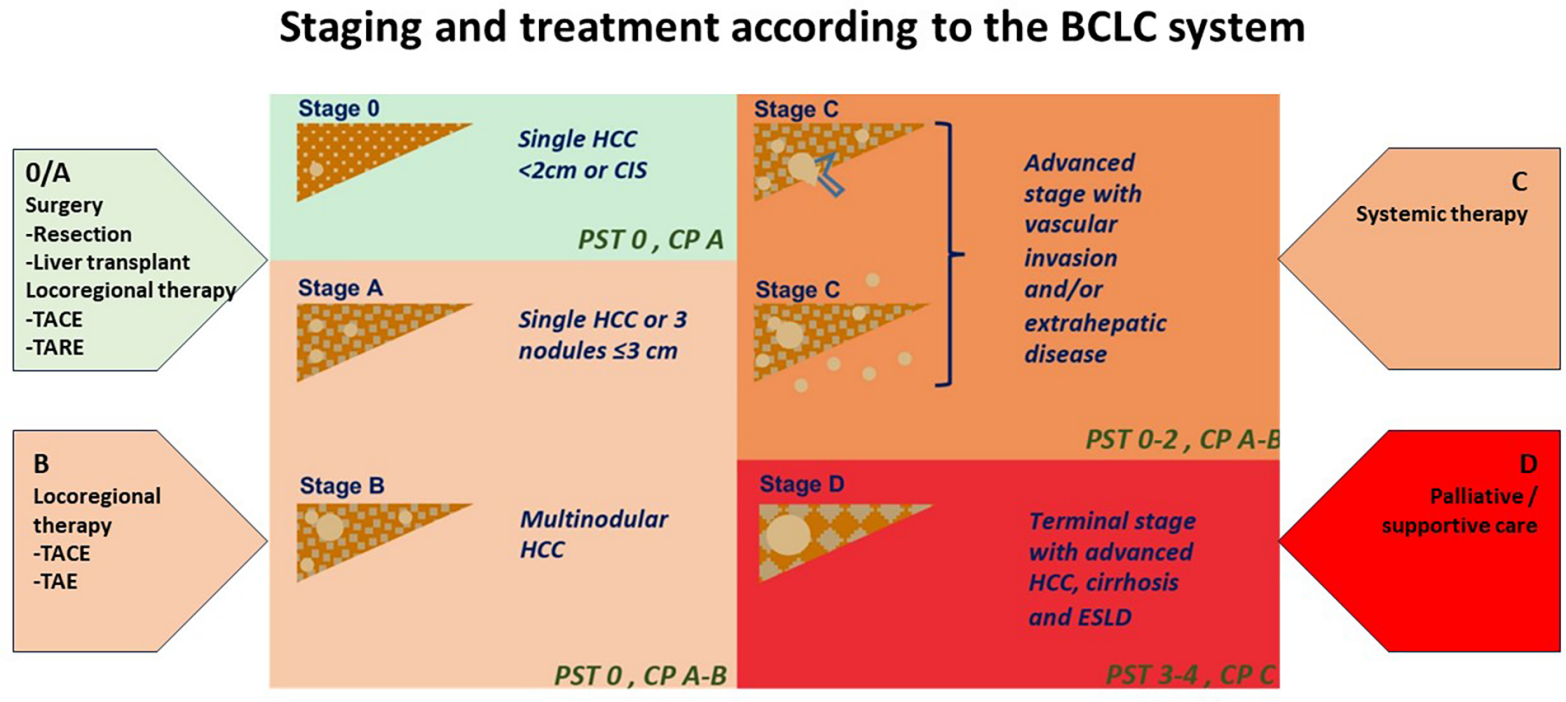
Staging and treatment based on the BCLC system. PST-performance status; CP-Child Pugh CIS-Carcinoma in situ HCC-hepatocellular carcinoma. Image adapted from *Int J Mol Sci*. (2019) 20:1465. doi: 10.3390/ijms20061465 *licensed under Creative Commons Attribution 4.0 International*.

Hepatocellular carcinoma (HCC) is the most common liver cancer, representing up to 75%–85% of primary cancers in the liver, and the third leading cause of cancer death worldwide (1, 2). HCC is a complex and heterogeneous disease with varying underlying aetiologies, risk factors and clinical manifestations. This tumor type tends to have a high propensity for growth due to its strong angiogenic activity and rich blood supply which play a critical role in tumor growth and metastasis. The treatment options for liver cancer have increased according to the Barcelona Clinic Liver Cancer (BCLC) algorithm guidelines (Figure 1). However, because HCC usually occurs in the background of underlying liver dysfunction and other comorbidities, it is often diagnosed late, and only approximately 30% of patients are eligible for curative treatments (i.e., resection, percutaneous ablation, etc.) at diagnosis (2–4). Prognosis can be generally poor despite a large range of treatment options.

Treatment options range from therapies that offer complete remission in early disease to supportive treatment in patients presenting late. Liver transplantation, partial liver resection and ablation offer a high rate of complete response dependent on early detection and management (3, 5). The BCLC staging algorithm can determine these treatment options (Figure 1) according to liver function, patient performance status and tumor burden. These may therefore include curative options usually reserved for early-stage disease (BCLC stage 0 to stage A). Liver resection is the therapy of choice in early disease; however, only a minority of patients fulfil the criteria for resection surgery or liver transplantation. Those with intermediate and advanced stage disease (BCLC Stage B and C) may be candidates for systemic therapy for liver metastases, transarterial chemoembolization (TACE), and radioembolization [i.e., selective internal radiation therapy (SIRT)] when surgery is not an option. Systemic therapy involves a combination of immunotherapy and anti-angiogenic therapies that may be limited by their toxic effects on the patient. In contrast, patients with end-stage disease (BCLC stage D) can only receive palliative care (3, 6, 4).

The efficacy of existing therapies varies widely depending on the patient's individual characteristics and disease status.

HCC tumors are typically hypervascular and therapy aimed at tumor vessel occlusion is hypothesized to induce hypoxia. This may contribute to the angiogenesis of HCC through inducing hypoxia, leading to the escape of HCC cells and thus subsequent chemo-and radioresistance, which are complex in mechanism (7, 8). Combination therapy of TACE or TAE with anti-angiogenic therapy has been used as an alternative strategy but has not provided complete success.

In recent years, theragnostics has emerged as a promising field that integrates diagnosis, therapy, and monitoring of therapeutic response into a single integrated process to enable personalised treatment strategies that can potentially improve patient outcomes. The field of HCC theragnostics using imaging and therapeutic radiopharmaceuticals is rapidly evolving, with new technologies and approaches being continually developed. Ideally, theragnostics would involve chemically and biologically identical compounds with adequate binding affinity allowing the diagnostic moiety and their therapeutic counterparts to have the same biodistribution when attached to a targeting moiety. Unfortunately, in some cases, the subtle differences in their chemical structures can affect their biological properties. A pair of theragnostic molecules which are not chemically or biologically identical may still adequately predict the biodistribution due to having similar enough biodistribution (9).

In HCC, the heterogeneity of tumors may pose a challenge in radionuclide therapy applications, making it important to consider factors such as the mechanism of localization of the theragnostic moieties, the retention time of the therapeutic moiety in these tumors, and also the time lapse between administrations noting the aggressiveness and extent of these tumors (9, 10). Low tumor uptake and rapid wash-out may result in rapid excretion making the treatment ineffective.

This paper explores the available and potential theragnostic approaches and the future of these patient-targeted treatment modalities in HCC.

### 1.1. Transarterial radionuclide therapy (TART)

HCCs are hypervascularised tumors mainly supplied by the hepatic artery, while normal liver tissue sees 80% of its supply coming from the portal vein (11, 12). This unique vascular anatomy allows for the administration of transarterial radionuclide therapy via the hepatic arterial route, resulting in a high absorbed dose delivered to the lesions and relatively low absorbed dose to the normal parenchyma. The intra-arterial delivery of treatment directly to the tumor is through methods such as transarterial chemoembolization (TACE) and selective internal radiotherapy (SIRT) or transarterial radioembolization (TARE). TARE is administered as an option for palliation or for patients who need tumor downstaging for additional interventional treatment (11).

Radioembolization is defined as the injection of micron-sized embolic particles loaded with a radioisotope by the use of percutaneous intra-arterial techniques (13). The use of intra-arterial radioactive compounds has multiple advantages. These include the ability to deliver high doses of radiation to small target volumes, the relatively low toxicity profile, the possibility to treat the whole liver, including microscopic disease, and the feasibility of combination with other therapy modalities (13). Radiopharmaceuticals used for TARE embolize the feeding artery and disseminate radiation, which destroys tumor cells. Following administration, the microspheres settle in the tumor and do not affect the vasculature, nor do they cause vessel occlusion. Patients can be discharged within a few hours of the procedure, be treated as an outpatient resulting in potentially improved quality of life (14).

These radioisotopes also allow for imaging either using bremsstrahlung or positron generation, as is the case with Ytrium-90 (^90^Y), or may emit gamma emission in addition to the therapeutic beta emission, allowing for additive diagnostic information which may guide the course of treatment (15). Imaging prior to and after administration of TARE is necessary to assess the arterial supply with their associated variations and the radiopharmaceutical distribution. Depending on the radiopharmaceutical administered, both positron emission tomography (PET) and single photon emission computed tomography (SPECT) imaging are optional. Before treatment, imaging helps to decide on the method of delivery, treatment dose and the precautions to take in cases of tracer deviations to other organs which may be unduly irradiated in the process. Imaging after injecting the theragnostic radionuclide into the hepatic artery helps to verify treatment delivery, the biodistribution of the radiopharmaceutical, radiation dose to the tumor as well as the dose exposed to the other organs of interest such as the lung, liver and stomach, as well as for the prediction of the treatment outcome (15). Further imaging can also be done to assess response, monitor the patient for complications that may occur, and plan additional therapies. Theragnostic applications in TART are thus quite beneficial in the customization of treatment to ensure the best outcome for the patient.

#### 1.1.1. Yttrium-90 (^90^Y) microspheres

Yttrium-90 (^90^Y) is a pure β emitter with energy of 0.937 MeV and penetration of 2.5 mm. It has a half-life of 64.2 h. These characteristics make it ideal for TARE. The lack of ɣ emission does not omit this radionuclide as a theragnostic radionuclide because bremsstrahlung SPECT imaging allows for assessment of the distribution of the tracer after administration. It is also possible to assess the placement of the ^90^Y labelled microspheres using PET imaging due to the internal pair production generated during the decay of this radionuclide. There are currently two commercially available ^90^Y-microspheres which are approved for administration, the 25-*μ*m glass microspheres (TheraSphere) and the 35-μm resin microspheres (SIR-Spheres) (16). Pre-administration imaging prior to ^90^Y labeled microspheres is only available through the administration of Technetium (^99m^Tc) labeled macro-aggregated albumin (MAA), which is a surrogate of ^90^Y microspheres. This is a disadvantage only in cases where there is discordance in the distribution of ^99m^Tc MAA and ^90^Y microspheres, as reported by Haste et al. (17). This discordance is hypothesized to be due to the different size, shape, and number of MAA particles compared to microspheres, flow dynamics during delivery and possibly the post-therapy activity.

Salem et al. reported long-term outcomes of 291 patients treated with ^90^Y radioembolization in a longitudinal cohort study. Response rate and time to progression (TTP) varied based on the Child–Pugh score of the patients. Patients with Child–Pugh A (indicating less severe liver disease) showed a survival of 17.2 months vs. the B group (indicating moderately severe disease) of 7.7 months; *P* = .002. Toxicities noted included fatigue in 57% of patients, pain in 23%, nausea/vomiting in 20% and grade 3/4 bilirubin toxicity in 19% of the cohort. The 30-day mortality rate was noted to be 3% (18). Yang et al. went on to prove that in patients treated with radioembolization with ^90^Y microspheres, the overall survival and response of the tumor was significantly improved compared to that of patients treated with chemoembolization (19).

#### 1.1.2. ^131^I-Lipiodol

Lipiodol is an iodinated and esterified lipid of poppy seed oil that has been used as a contrast agent for detecting liver cancer. It is the most effective and convenient carrier because of its excellent targeting ability and capacity to be accurately monitored by x-ray (20). It is an oily medium that is selectively retained in the tumor when administered intra-arterially (21). Mechanisms postulated for this retention include an embolization effect, the presence of abnormal tumor vessels, abnormal tumor blood flow, a lack of macrophages or a lack of lymphatics in the tumor and rapid active uptake of Lipiodol by HCC cells through phagocytosis into the liver cell (22, 23). Iodine-131 (^131^I) labelled lipiodol is commercially available for the treatment of liver cancer. ^131^I emits both β and ɣ rays. The β particles have a maximum energy of 0.6 MeV and a maximum tissue range of 2.3 mm. ^131^I-lipiodol selectively accumulates in hepatocellular carcinoma with prolonged retention compared to normal liver parenchyma tissues and with minimal irradiation damage being reported despite the normal more homogeneous accumulation described (24–26). The long half-life of 8.04 days meant that on imaging, ^131^I-lipiodol could be noted in the liver tumors even after seven days of administration. Unfortunately, reports of prolonged accumulation have also been noted in the normal liver and the lungs, indicating that toxicity to these structures was likely (24).

Lipiodol has been labeled with iodine-131 (^131^I), rhenium-188(^188^Re), yttrium-90(^90^Y), holmium-166 (^166^Ho), and lutetium-177(^177^Lu) (27).

Lintia-Gaultier et al. reported their 7 years' experience with ^131^I-Lipiodol. The efficacy of intra-arterial radioembolization compared with patients not receiving this treatment showed 32 weeks median survival compared with 8 weeks for the untreated group (*P* = 0.007). This group did not report any radiotoxic effects in the treated group, indicating that ^131^I-Lipiodol is safe and provides significant overall survival for patients with advanced HCC (26). Ahmadzadehfar et al. also reported a longer overall survival in patients with Child-Pugh A in those treated with ^131^I Lipiodol (28). Undesirable side effects reported with ^131^I-Lipiodol include fever, moderate and temporary disturbances of the biological liver test, pain on injection and rarely leukopenia and serious diffuse infiltrative pneumopathies (22). However, in comparison with chemoembolization, Bhattacharya et al. reported only three serious side-effects vs. 29 on the chemo-embolization arm (29).

#### 1.1.3. Rhenium-188 (^188^Re) /lipiodol complex for transarterial liver cancer therapy

Another well-studied radioisotope for transarterial radionuclide therapy for liver cancer is Rhenium-188 (^188^Re).^188^Re labelled lipiodol is a promising radiotherapy agent due to the maximum β emission energy of 2.1 MeV, which is responsible for the destruction of tumor tissue. Its maximum range in tumor tissue is up to 10.1 mm, which is larger than that of ^131^I (2.4 mm) and makes it very effective in tumor destruction. Although the range is comparable with ^90^Y (10.8 mm), unlike ^90^Y, it has 155-keV ɣ emissions, making imaging for biodistribution studies and external dosimetry possible. Its short physical half-life and the fact that most ɣ emissions are at 155 keV only, results in very low radiation exposure to relatives and hospital staff (30). The other advantages of using ^188^Re for radionuclide therapy include its convenience, that it is inexpensive, and the onsite availability from the tungsten-188 (^188^W)/^188^Re generator (31–36).

Various types of ^188^Re-related preparations in clinical research exist, using different chelators for labeling. The most widely studied compound is ^188^Re-labeled 4-hexadecyl-1,2,9,9-tetramethyl-4,7-diaza-1,10-decanethiol/lipiodol (^188^Re-HDD lipiodol). Unfortunately, the *in vivo* stability of this complex is not optimal (37).

##### 1.1.3.1. ^188^Re-HDD lipiodol

Most of the clinical trials and studies, including the International Atomic Energy Agency (IAEA)-sponsored multicentre study, have used these HDD kits in the preparation of ^188^Re-Lipiodol radio-conjugate.

Results of a few clinical studies involving multiple or single centres, have already been published, highlighting the efficacy of ^188^Re Lipiodol in the treatment of HCC. Most studies have used the ^188^Re-HDD Lipiodol radio conjugate for the treatment. However, Boschi et al. used bis-(diethyldithiocarbamato) nitrido (N-DEDC), ^188^Re-N-DEDC Lipiodol complex. Lambert et al. performed preliminary feasibility studies in Belgium and Asia and confirmed the radio-conjugate's safety and tolerability in patients suffering from HCC (38, 39).

The IAEA-supported multicentre and multinational trial is probably the most important study conducted so far using ^188^Re Lipiodol radio-conjugate, and it was unique in the sense that the study was conducted using a single protocol utilising a standard labeling procedure and dosimetry methodology in eight countries across two continents (40).

Results of the phase I and preliminary results of the phase II trials sponsored by the IAEA have shown TART with ^188^Re-HDD iodised oil to be safe and effective in patients with HCC, which is similar to the initial experience by Sundram et al. (41, 42). In the Phase I clinical trial, 70 patients received at least one ^188^Re-HDD lipiodol treatment, and the results showed a median survival of 9.5 months (42). The Phase II clinical trial results of the study, published in 2007, showed that of the 185 patients from 8 countries who received the ^188^Re-iodine oil treatment, the 1-year and 2-year survival rates were 46% and 23%, respectively, with an observed good tolerance (43). Kostas Delaunay et al. conducted a Phase I study of ^188^Re-SSS lipiodol to treat HCC. The results show that ^188^Re-SSS lipiodol (perthiobenzoate and dithiobenzoate moieties [M (PhCS_3_)_2_ (PhCS_2_)] nicknamed SSS, standing for “Super-Six sulphur”) has a good biodistribution in radioactive embolism. Of the radiolabeled lipiodols reported to date, it is the most stable in the body (44).

Transarterial radionuclide therapy (TART) with ^188^Re-lipiodol appears to be a safe, effective, economical and promising therapeutic option in patients with inoperable large and/or multifocal hepatocellular carcinoma (41, 42).

#### 1.1.4. Lutetium-177 labelled radiopharmaceuticals for transarterial liver cancer therapy

Lutetium-177 (^177^Lu) is a well-known and effective theragnostic radionuclide. It has a half-life of 6.7 d, which is found to be highly suitable as a therapeutic radionuclide, and releases medium-energy photons of 113 and 208 keV respectively, which are used for diagnostic imaging (45, 46). ^177^Lu radiopharmaceuticals are not yet approved in their role of TARE in HCC, however, a few radiopharmaceuticals have been created in this scope.

Chan et al. successfully developed chitosan microspheres radiolabeled to the theragnostic pair, Indium-111 and ^177^Lu to form ^111^In/^177^Lu-DTPA-CMS with radiochemical yields greater than 90% and high radiochemical purities (>98%). Chitosan is derived from the deacetylation of chitin and is known to have a high biocompatibility with low cytotoxicity. These microspheres have a diameter of 36.5 ± 5.3 micrometres which are ideal for TARE. They noted good retention in HCC with only 1% radioactivity being distributed to normal organs. They were also able to demonstrate a significant reduction in the size of the HCC lesions of the rats 10 days after injection of ^177^Lu-DTPA-CMS while the lesions in the control group got larger. This makes ^111^In/^177^Lu-DTPA-CMS a potentially superior theragnostic pair for the TART of HCC, and with the imaging characteristics of ^177^Lu, this gives the added advantage of performing dosimetry and tracking the distribution after administration.

Another experimental ^177^Lu-labeled radiopharmaceutical for TART is the polydopamine (PDA)-coated ^177^Lu-radiolabeled silica microspheres (^177^Lu-MS@PDA). ^177^Lu-MS@PDA is developed by Wu et al. who demonstrated prolonged retention in the HCC lesions and excellent radiostability of the radiopharmaceutical. These microspheres showed good biocompatibility, had a large specific surface area, good ion absorption capacity, and are good drug carriers making them ideal for this type of intervention (46).

## 2. Potential theragnostic radionuclide therapies for hepatocellular carcinoma

In recent developments, several imaging biomarkers have shown a clear advantage in detecting hepatocellular cancer. In particular, the theragnostic potential of radiolabeled prostate-specific membrane antigen (PSMA) and fibroblast activation protein inhibitors (FAPI) in various malignancies is encouraging as the potential for translation to theragnostics in HCC seems like a great possibility.

Radiolabeled PSMA and FAPI, mainly with Gallium-68 (^68^Ga), have demonstrated a clear advantage in imaging of HCC compared to the more commonly used 18F-FDG. In some cases, they, have even shown higher sensitivity and accuracy than conventional imaging with CT and/or MRI, especially for metastatic disease (47). Investigating these ligands for potential theragnostic application, seeing if they can be labeled successfully to the therapeutic radionuclides, is worth exploring. To do this, the kinetics of these radiopharmaceuticals would need to be evaluated.

### 2.1. Ga-68 PSMA

PSMA is a glutamate carboxypeptidase II transmembrane glycoprotein that is a zinc mettaloenzyme. It catalyses the hydrolysis of N-acetylaspartylglutamate to glutamate and N-acetylaspartate (48). Its overexpression in prostate cancer has been studied extensively and has been validated for theragnostic use in metastatic castrate-resistant prostate cancer.

The overexpression of PSMA is not only confined to the prostate, but accumulation in non-prostatic solid tumors such as glial, gastrointestinal, urothelial, hepatocellular, pancreatobiliary, lung, breast, thyroid, and renal neoplasms amongst other tissues has also been reported. The high accumulation of PSMA in non-prostatic malignancies is found to be associated with overexpression on neovasculature formation in the tumor (49–51).

Morphological imaging plays an important role in contrast-enhanced computed tomography (CT) and magnetic resonance imaging (MRI) based on the Liver Imaging and Reporting Data System (LI-RADS) criteria. Still, little insight is provided about the biology of HCC, and the biology of the tumor is important, especially in theragnostics (48).

A biopsy is another form of assisting with the diagnosis, but it also presents several limitations, namely intratumor heterogeneity, sampling errors and difficulty distinguishing between early-stage HCC and dysplastic nodules (52). Molecular imaging is an imaging modality that is being explored and may provide additive information that may lead to a theragnostic era for HCC. Preclinical studies report PSMA overexpression in the vascular endothelium of HCC, resulting in increased accumulation in this malignancy (53, 54). This kinetics opens the stage for assessing the avidity of this agent and its possibility of application in therapy.

The most commonly used radiotracer for PET imaging in malignancies is fluorine-18fluorodeoxyglucose (^18^F-FDG), and it has demonstrated suboptimal liver imaging kinetics and ^18^F-FDG avidity is mostly visualised in a fraction of high-grade tumors. Even with the addition of radiolabeled choline with ^18^F or carbon-11(^11^C), in early HCC there was no demonstratable benefit as it has a low diagnostic accuracy (<40%) (55, 56). Recent studies comparing ^68^Ga PSMA with either ^18^F-FDG have demonstrated the superiority of ^68^Ga PSMA in intrahepatic as well metastatic detection of HCC (48, 57, 58).

PSMA uptake in the hepatic lesions is evaluated qualitatively and semi-quantitatively on a whole-body survey using positron emission tomography/ computed tomography (PET/CT) scan. The benefit of pursuing PSMA imaging will be to assess eligibility for PSMA-targeted therapy (6, 48, 58, 59). Clinical validation of PSMA-directed radioligand therapy (RLT) with Lutetium-177 (^177^Lu) -PSMA is ongoing, and the safety and efficacy of this therapy has been established in prostate cancer treatment. In patients with HCC, limited scientific data is currently available.

**Figure 2:**
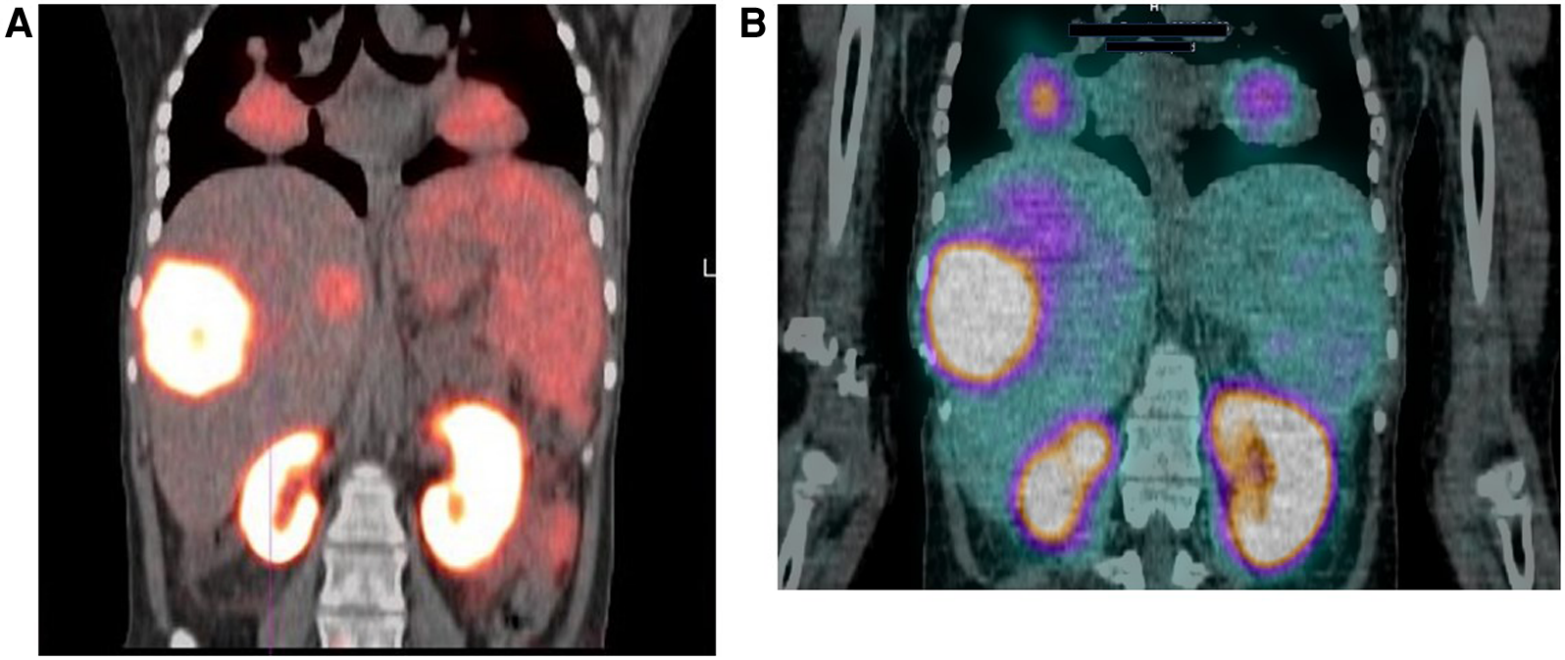
18-year-old female with a 2-year history of HCC. The patient had a hepatectomy and subsequent recurrence, which was not responding to conventional therapy. (**A**) Ga-68 PSMA scan demonstrating uptake in the liver and lung lesions. (**B**) Lu-177 PSMA post-therapy image, demonstrating congruent uptake in these lesions.

**Figure 3:**
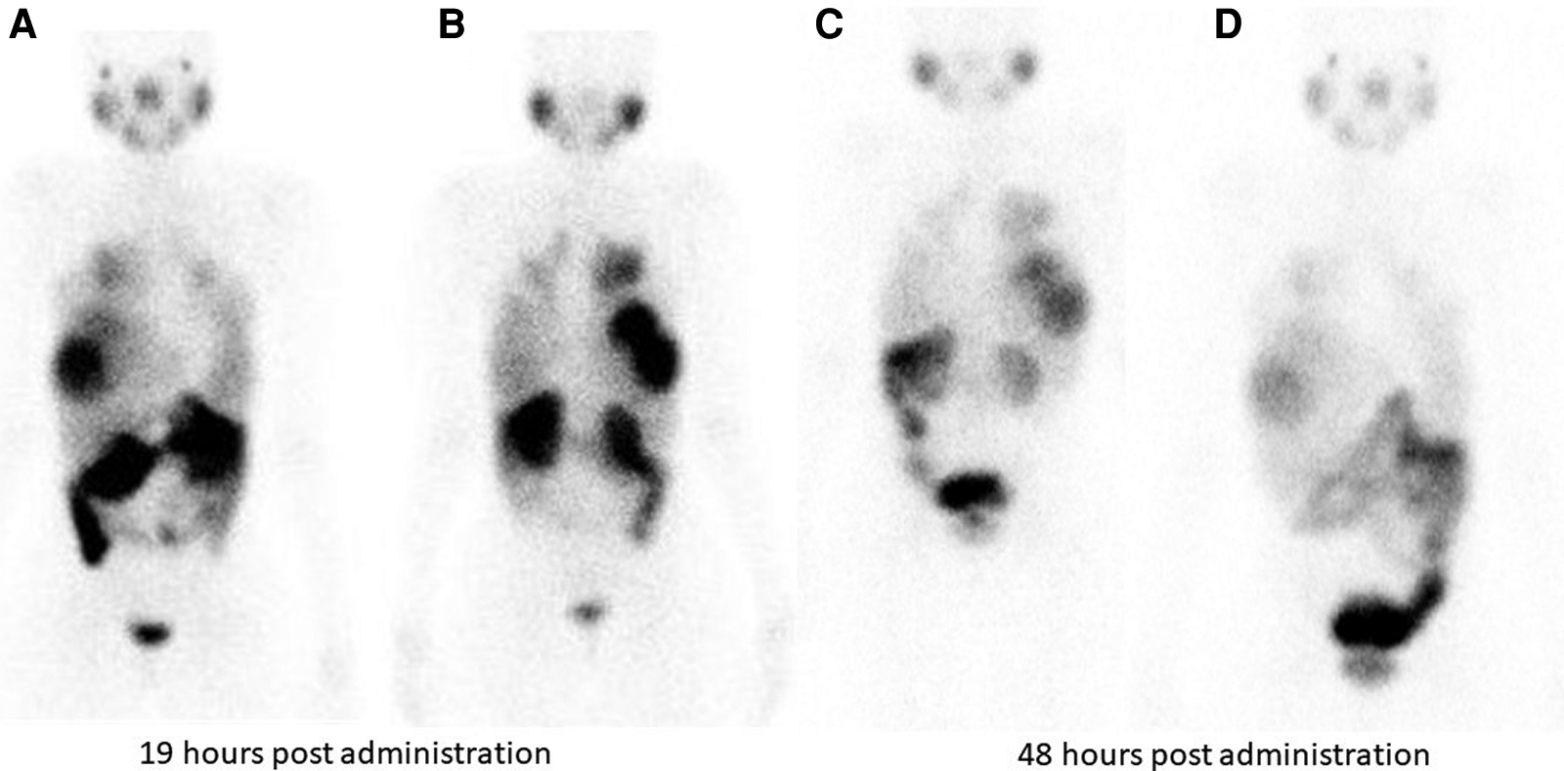
Tracer retention in the liver lesions is seen at 19 h and 48 h following ^177^Lu PSMA administration in patient shown in Figure 2. (**A**) (anterior) and (**B**) (posterior) are taken 19 hours post Lu-177 PSMA administration. Images (**C**) (anterior) and (**D**) (posterior) are taken at 48 h post Lu-177 PSMA administration.

Hirmas et al. administered one cycle each of ^177^Lu PSMA-11 in two patients, respectively. The radiation dose in the liver lesions was found to be low, and thus the treatment was deemed ineffective (6). We administered ^177^Lu PSMA therapy in a patient who had no remaining treatment options and could only benefit from palliation. There was evident and intense accumulation of ^177^Lu PSMA in the lesions, which was congruent to the ^68^Ga PSMA biodistribution noted prior (Figure 2). The retention in the lesions was still noted up to 48 h of imaging post treatment infusion on the post therapy ^177^Lu PSMA imaging (Figure 3). This indicates a need for proper evaluation of the kinetics of this radioligand in HCC. Low uptake and rapid wash-out from the tumor possibly related to rapid excretion and low binding affinity of low-molecular-weight ligands are hypothesized as potential issues affecting the efficacy of RLT in HCC.

In a preclinical study by Lu et al, the radioligand ^177^Lu-Evans blue (EB)-PSMA-617 was compared to ^177^Lu-PSMA-617. There was evidence of higher accumulation, longer retention time with slower clearance from the tumor when using ^177^Lu-EB-PSMA-617 compared to ^177^Lu-PSMA-617 which exhibited a short residence time in the tumor (10). This group also assessed survival comparing both radioligands with a control group treated with saline. Both radioligands demonstrated improved survival compared to the control group. The tumor size was suppressed in both treatment groups when compared to the control group and no healthy organ toxicity was observed with either intervention (10).

The more advanced the stage of disease, the poorer the prognosis for the patient. The first line of therapy is usually a combination of immunotherapy and anti-angiogenic therapy, but the toxicity effects of the treatment limit most patients.

With the limited treatment options available for this patient population, the availability of RLT offers a safe and selective delivery of the targeted therapy, reduced toxicity as treatment doses are individualized, and improved outcomes for the patients are expected.

### 2.2. Radiolabeled FAPI in liver tumors

Molecular-based imaging and therapeutic strategies include targeting the tumor microenvironment. Among the targets is fibroblast activation protein-α (FAPα), which is overexpressed on CAF (cancer-associated fibroblasts). CAF are present in the stroma of approximately 90% of malignant epithelial neoplasia (60, 61). FAPα can be targeted with radiolabeled (RL) FAPIs (fibroblast activation protein inhibitors). Several FAPI ligands have been described in the literature: AIF-FAPI-74 is solely for diagnostic use and has been labeled with ^68^Ga and ^18^F; the remaining FAPI molecules incorporate either a DOTA or DOTAGA chelate and are labeled with ^68^Ga. These chelating agents allow tagging with therapeutic radioisotopes such as ^177^Lu, ^90^Y and ^225^Ac, which broadens the clinical repertoire of RL FAPI from diagnostic to theragnostic (62).

^18^F-FDG is currently the most widely used radiotracer for molecular imaging in oncology. However, the diagnostic accuracy of FDG studies is hampered by its wide normal biodistribution, which reflects glucose utilization in normal organs, including the liver (the major producer of nondietary glucose) (63). Consequently, normal liver parenchyma demonstrates a mild to moderately intense, uniformly mottled appearance, and it may be difficult to distinguish increased focal pathological uptake from normal background physiological activity. This may lead to lower diagnostic accuracy, particularly for low-grade and smaller liver lesions (64). Furthermore, the quality of FDG studies is also dependant on adequate preparation: patients are required to fast and follow a low-carbohydrate diet (to ensure euglycaemia at the time of FDG injection) to prevent competitive inhibition of FDG uptake and altered biodistribution, both of which may further complicate diagnostic interpretation (65).

The growing interest in radiolabeled FAPI as a general oncological molecular probe is mainly due to its more favourable biodistribution. FAPI uptake is independent of glucose metabolism with resultant significant reduction of background activity in the brain, oro- and nasopharyngeal mucosa, gastrointestinal tract and liver (60). PET FAPI thus allows for high image quality with significantly better tumor-to-background ratios (TBR) and improved contrast resolution compared to FDG PET (66). Furthermore, patient preparation is less stringent, and ^68^Ga-FAPI can be used without specific dietary preparation (67).

In a highly cited pioneering paper, the Heidelberg group reported 68Ga-FAPI uptake in 28 different cancers, including small-volume liver metastases (68). In a retrospective study Wang et al. compared ^68^Ga-FAPI-04 and ^18^F-FDG PET/CT in detecting hepatocellular carcinoma (HCC) in 29 patients (69). They found that ^68^Ga-FAPI-04 PET/CT was significantly more sensitive than ^18^F-FDG PET/CT in detecting intrahepatic HCC lesions (85.7% vs. 57.1%, *P* = 0.002). Sensitivity was preserved for smaller (≤2 cm in diameter; 68.8% vs. 18.8%, *P* = 0.008) and well- or moderately differentiated (83.3% vs. 33.3%, *P* = 0.031) tumors. This study did not find a statistically significant difference in SUVmax between the two tracers, but TBR was significantly higher in the ^68^Ga-FAPI-04 group compared with the ^18^F-FDG group (11.90 ± 8.35 vs. 3.14 ± 1.59, *P* < 0.001). Furthermore, a significant correlation was observed between tumor size and SUVmax & TBR in ^68^Ga-FAPI-04 positive lesions (*P* < 0.05). Similarly, Shi and colleagues (70) prospectively evaluated primary intrahepatic tumors with ^68^Ga-FAPI-04 and ^18^F-FDG. In 17/20 patients [14 with HCC and 3 with intrahepatic cholangiocarcinoma (ICC)], they found that the diagnostic sensitivity of ^68^Ga-FAPI (sensitivity: 100%, specificity: 100%) was significantly higher than that of ^18^F-FDG avid (sensitivity: 58.8%, specificity: 100%). Furthermore, not only were SUVmax and TBR values comparatively higher on the ^68^Ga-FAPI PET/CT, but a larger number of extrahepatic metastases were detected with FAPI as well. Benign liver lesions were diagnosed in 3/20, and all had negligible uptake in both studies. Guo et al. published a retrospective, single-centre review of 34 patients with known or suspected primary liver tumors. They compared the diagnostic performance of PET/CT with 68Ga-FAPI and 18F-FDG as contrast-enhanced CT (CECT) and MRI (71). In the cohort, 20/34 had HCC, 12/34 were diagnosed with ICC and the remaining 2/34 patients presented with benign hepatic nodules. The sensitivity of ^68^Ga-FAPI (96%) was similar to that of MRI (98.1%) and was once more significantly higher than that of ^18^F-FDG (65%). Although less sensitive than CECT (100%) for intrahepatic lesions, ^68^Ga-FAPI whole body PET/CT demonstrated added value by detecting malignant lesions in extrahepatic sites with high sensitivity. In an interesting paper on evaluating tracer kinetics following dynamic PET/CT imaging with ^68^Ga-FAPI, the authors reported statistically significant differences in several kinetic parameters to distinguish between HCC, non-HCC lesions, and healthy liver tissue.^13^ In a more recent publication, Zhang et al. prospectively studied the utility of ^18^F-FAPI in 37 patients with non-FDG avid focal liver lesions (FLL). The sensitivity, specificity, and accuracy of ^18^F-FAPI were 96.0%, 58.3%, and 83.8%, respectively. On semiquantitative analysis, they report that the SUVmax and TBR were significantly higher in malignant vs. benign FLLs.

In evaluating intrahepatic lesions with FAPI PET, false positives have been described in chronic hepatitis, cirrhosis, active liver fibrosis, focal nodular hyperplasia, hepatic adenomas, inflammation and post-surgical fibrosis/wound healing. False negatives are likely in lesions smaller than the spatial resolution of the PET camera and tumors with low CAF expression.

Targeting the tumor microenvironment with FAPI radionuclide therapy represents a novel therapeutic strategy. As mentioned, chelators allow labeling various FAPI ligands with therapeutic radioisotopes. Comparative studies have identified FAPI-04 and FAPI-46 as the most suitable, currently available compounds for theragnostics due to high stability in human serum, high specific binding affinity to FAP and higher cellular retention. FAPI-46 has the added advantage of more prolonged retention/slower washout (72).

Current data on theragnostic applications of FAPI are based on single institution proof-of-concept studies in patients with a variety of end-stage cancers and, to date, no FAPI based treatment has been carried out in intrahepatic lesions specifically (62, 73).

Since the first clinical applications were described in 2019, there has been growing interest in both the diagnostic and theragnostic applications of RL FAPI. While early data is encouraging, more robust clinical evaluation and validation are still required.

## 3. Conclusion

Theragnostics in HCC offers a personalized management style which is needed in HCC treatment given the rate of recurrence and the common late presentation of patients which leads to a limitation in the treatment options. The treatments available are shown to be safe and effective and promise a solution in inoperable disease. Further investigations of the newer and potential systemic radionuclide therapy could thus benefit this population in the future. Alpha emitting and Auger electron radionuclide therapies, with their high linear energy transfer, could also be considered in transarterial radiotherapy to circumvent the prospect of radio resistance and chemoresistance induced through hypoxia of these tumors from conventional treatment methods.
